# Experiences of cervical cancer screening in HIV-positive women in Zimbabwe

**DOI:** 10.4102/curationis.v44i1.2184

**Published:** 2021-11-10

**Authors:** Patience C. Mpata, Zethu Z. Nkosi

**Affiliations:** 1 School of Nursing and Midwifery, Mpilo Central Hospital, Bulawayo, Zimbabwe; 2 College of Human Sciences, University of South Africa, Pretoria, South Africa

**Keywords:** adopters and non-adopters of cervical cancer screening, cervical cancer, facilitators, focus group discussions screening, HIV, human papilloma virus, in-depth interviews, qualitative study

## Abstract

**Background:**

The primary purpose of screening is to detect individuals in danger of cervical cancer so as to prevent further progression of the disease. Cervical cancer remains a global concern, as it ranks as the fourth most commonly diagnosed female malignancy worldwide. It is the commonest female cancer in Zimbabwe. Women living with human immunodeficiency virus (HIV) have a disproportionate risk of invasive cervical cancer, as they are 2–12 times more likely to develop pre-cancerous lesions. As a result of the increased risk, routine screenings are suggested. Few women are screened for cervical cancer in Zimbabwe.

**Objectives:**

This study aimed at describing the experiences of screening for cervical cancer and motivation behind screening.

**Method:**

The study employed a qualitative research approach. In-depth one to one interviews and focus group discussions were conducted using interview and focus group guides. The study was conducted at an opportunistic infections clinic in Mpilo Central Hospital. Data analysis was performed by using Giorgi’s descriptive method of data analysis.

**Results:**

The themes that emerged from data analysis were facilitators to screening for cervical cancer, community awareness of cervical cancer screening, free cervical cancer treatment and more screening centres and integrating cervical cancer screening with HIV care.

**Conclusion:**

In-depth understanding of the factors that enable women to take part in cervical cancer screening is essential so that these factors can be strengthened to improve uptake of cervical cancer screening services.

## Introduction

Cervical cancer is ranked as the fourth most commonly diagnosed female cancer globally (Bray et al. 2018:394) with an estimated 311 000 deaths, of which more than 85% of these deaths occur in low- and middle-income countries (WHO 2017). In Zimbabwe cervical cancer is the most frequently diagnosed female cancer and accounts for 33.9% of the cancer burden (Minister of Health and Child Welfare – Zimbabwe National Cancer Strategy 2013–2017). Annually 3186 women are diagnosed with cervical cancer and 2151 die from the disease (World Health Organization Information Center on Human Papilloma Virus and cervical cancer [WHO/ICO HPV] 2016), if no interventions are put in place the numbers are expected to increase. However, data indicate that in the last three decades there has been a decline in cervical cancer rates in most of the developed countries, as compared with the developing nations. It has been observed that countries with well organised screening programmes, rates of mortality and morbidity have declined significantly (WHO/ICO 2010).

Aetiology of cervical cancer is well understood enabling prevention and cure. Cervical cancer is curable if diagnosed early (Subramanian et al. 2016:4). The 10-to-20-year lag between the pre-cancer and invasive cancer allows an opportunity to screen and detect the disease before its progression to invasive cancer; however, in women with weakened immune systems such as those with untreated human immunodeficiency virus (HIV) it can take only 5–10 years for invasive cancer to develop (WHO 2017).

Women living with HIV are more prone to human papillomavirus (HPV) infection and should therefore be followed up closely for evidence of precancerous changes (WHO 2017). In Zimbabwe the prevalence of HPV infection was found to be higher (54%) in HIV-positive women than (27%) in HIV-negative women (Gundani & Chipfuwa 2013:26).

According to the United Nations Programme on HIV and AIDS (UNAIDS 2019) Zimbabwe has one of the highest prevalence rates in sub-Saharan Africa at 12.8%, with an estimated 730 000 women living with HIV. It is suggested that HIV-positive women should undergo more frequent screenings for cervical cancer as they are more likely to suffer from the disease. The World Health Organization (2013:10) recommended that in areas with high endemic HIV infection, women who are HIV-positive, the repeat screening interval should be within three years if the screening test is negative.

Various methods of screening are used, which include Papanicolaou (Pap) smear, visual inspection with acetic acid and cervicography (VIAC) and human papillomavirus deoxyribonucleic acid (HPV DNA) testing. Zimbabwe adopted VIAC in 2012 and women can access it at six weeks post-delivery at no cost in public health centres. Visual inspection with acetic acid is referred to as screen-and-treat because results are immediately available and affords the woman a chance to be treated on a single visit (Kuhn & Denny 2017:4). Visual inspection with acetic acid is a relatively simple procedure performed by doctors and nurses in Zimbabwe.

In an effort to increase access to screening for cervical cancer, innovative strategies are emerging such as involvement of male partners, integration of screening services with HIV care and self-sampling by women (Li et al. 2017:18). Raising awareness about cervical cancer is important as it has been discovered that women can be motivated to participate in regular screening when they consider themselves to be at risk of cervical cancer, whereas women with gaps in knowledge have inadequate understanding of cervical cancer prevention process (Massad et al. 2015:37).

There is scarcity of information on what facilitates screening for cervical cancer in Zimbabwe. Therefore, knowledge of factors that would motivate women to take up cervical cancer screening is vital as the information could be used to develop interventions to enhance screening uptake. Hence, this study aimed to get a better understanding of the factors that facilitate cervical cancer screening in HIV-positive women.

## Problem statement

Regardless of the availability of cervical cancer screening services, the adoption rate remains very low despite the fact that public health facilities offer screening free of charge. The estimated coverage of cervical cancer screening in Zimbabwe is 7.2% in the general female population (Human Papillomavirus Centre 2016:61). In order to improve and implement user friendly programmes so as to increase and encourage screening, there is need to find out why there is low usage of the existing cervical cancer screening services. Hence, the study sought to explore women’s experiences of cervical cancer screening and the motivation to screen.

## Aim of the study

As a result of the limited understanding regarding dynamics associated with cervical cancer screening, the study’s aim was to describe the lived experience of screening for cervical cancer and what motivates uptake for cervical cancer screening in HIV-positive women in Zimbabwe.

## Methods

Qualitative research approach was used with a descriptive phenomenology design (Polit & Beck 2012:495). Descriptive phenomenology was used to explore, analyse and describe the experience of screening for cervical cancer so as to gain a near real picture by maintaining the richness and depth of the phenomenon (Matua & Van der Wal 2015:23; Streubert & Carpenter 2011:81). The study was conducted at Mpilo opportunistic infections clinic. In the Zimbabwean context, opportunistic infection clinic is where people knowingly living with HIV go for supply of antiretroviral medication. The population chosen for this study consisted of all HIV-positive women who are on antiretroviral treatment at one of the public hospitals in Zimbabwe. Using a purposive-criterion based sampling method that involves selecting participants who meet a predetermined criterion of importance (Polit & Beck 2012:519; Streubert & Carpenter 2011:28), information rich participants were selected to actively participate in the study by describing the experience of being screened for cervical cancer. The inclusion criteria included being HIV-positive, having been on antiretroviral treatment for more than a year, having screened for cervical cancer, having no hysterectomy and 18 years and above.

In-depth interviews were used together with focus group discussions to collect data, both methods were used so as to triangulate data. Twenty-two in-depth interviews were used to collect detailed accounts of participants’ thoughts, beliefs and knowledge pertaining to cervical cancer screening and expressions of their experiences reflected in reality (Lambert & Loiselle 2008:229). Four focus group discussions, two groups with three participants each and the other two with four participants each were conducted as a means to elicit more information and to discover variety of participants experiences with screening for cervical cancer (Fusch & Ness 2015:11). Pretested interview guides were used to collect data and probes were used to elicit further description of unclear points (Ritchie & Lewis 2003:168).

## Data collection

The study was conducted over four months from November 2017 to February 2018, at a purposively selected opportunistic infections’ clinic. Permission to access the site was obtained from the hospital authorities before commencing data collection. After information sessions by the researcher, voluntary participants gave written consent. They were made aware that they were at liberty to withdraw their participation at any stage of the study without being penalised. Demographic data forms were completed prior to discussions. Demographic data forms focused on age, level of education, marital status, parity and employment status. Interview guides were used for in-depth and focus group discussions. The interview guide had questions on knowledge about cervical cancer, views/experience on screening for cervical cancer, facilitators/barriers to screening and perception of risk. Data saturation was reached after 22 in-depth interviews and four focus group discussions comprising of adopters of cervical cancer screening. A digital recorder was used for audio taping information during interviews and focus discussions using the Ndebele language, which is spoken in the region. The information was then translated and transcribed. Computer files were generated and documents were stored in folders named appropriately. Codes were used to ensure anonymity, privacy and confidentiality. Only the researcher and supervisor had access to the information.

## Data analysis

Demographic data were analysed using percentage distribution and presented in tables. Interview and focus group discussion audios were transcribed verbatim, after which the researcher became immersed in the data. Giorgi’s phenomenology method of data analysis was used to have a better understanding of the lived experience of screening for cervical cancer (Polit & Beck 2012:567). The researcher read through the entire transcribed data to get a sense of the whole, data were broken down to units from participants descriptions, units were then combined to come up with themes (Whiting 2001:60).

## Results

The results of the demographic profile are presented in Table 1.

**TABLE 1:** Demographics of study participants (*n* = 36).

Variable	Frequency	Percentage
**Age**		
20–29	5	13.9
30–39	13	36.0
40–49	14	39.0
≥ 50	4	11.1
**Level of education**		
Primary level	8	22.2
Secondary level	27	75.0
Tertiary level	1	2.8
**Marital status**		
Married	19	52.8
Single	11	30.6
Widowed	5	13.9
Separated	1	2.8
**Parity**		
0–1	10	27.8
2–3	20	55.6
4–5	5	13.9
≥ 6	1	2.8
**Employment status**		
Unemployed	24	66.7
Employed	12	33.3

**Total**	**36**	**100.0**

## Facilitators to cervical cancer screening

Having signs and symptoms of cervical cancer facilitated screening in this study’s participants. Some women felt that being HIV-positive made them more vulnerable to cervical cancer therefore they were motivated to undergo screening. Support and encouragement from the spouse also played a role in facilitating screening. Other women suggested that forcing women to screen would be the best approach to follow in an attempt to have more women taking part in screening:

‘What motivates me is that I am HIV [*human immunodeficiency virus*]-positive and I am more prone to cervical cancer because I no longer have the immunity to protect me from various diseases, I get diseases easily.’ (IDI 1, interview 1, 37 years, married, 3 children)

‘It is my husband who encouraged me to go and screen, I also used to think that if I screened they would remove my womb, therefore I was afraid that they would tell me that my womb was not okay and they would want to remove it.’ (IDI 5, interview 5, 34 years, married, 2 children)

‘I just had pains, my womb was painful, at times when bathing I would insert my fingers in the vagina and at times would scrap around and would see a blood-stained discharge, I informed my husband who suggested that I should go for cancer screening at the hospital.’ (IDI 5, interview 5, 34 years, married, 2 children)

‘I feel that women should be forced to screen.’ (IDI 19, interview 19, 27 years, single, 2 children)

One participant highlighted that having young children motivated her to screen as she did not want to die, she wanted to stay safe for her children’s sake:

‘I was motivated by the situation that I am in. I have young children, so I do not want to die of cancer without even knowing where I got it from. One has to always know that they are on the safe side.’ (IDI 9, interview 9, 35 years, married, 4 children)

Access to information and having trust in the health delivery system motivated some women to be screened for cervical cancer:

‘… I had heard of a disease called cervical cancer and that women should go for screening so I prepared myself for that. For as long as there is something in hospital not just being done by someone else but by health workers it is important to take part as it may save one’s life. Nowadays there are too many illnesses so it is best to be careful.’ (IDI 17, interview 17, 31 years, married, 1 child)

## Community awareness of cervical cancer screening

Receiving information was identified as one of the main facilitators for making it easier to participate in cervical cancer. Participants highlighted that information received was insufficient they were only informed to avail themselves for screening without even knowing what causes the disease. Other women suggested that regular reminders would be helpful for continued participation:

‘I feel that if there should be people to teach us about cancer of the mouth of the womb. Especially in the morning before we get our treatment, they could also put some fliers or brochures by the roadside or places where most people go so that anyone who is interested can read about it. The other option would be to use radios or television to teach people about it, but with this difficult economic situation it is difficult to get time to listen to the radio or watch television.’ (IDI 8, interview 8, 45 years, widow, 3 children)

‘Women can be encouraged to screen by reminding and teaching them about cervical cancer screening, so that they become aware that there is such a problem; it should not come as a surprise. Some women are afraid of finding out that they have the disease. But it is good that one should know what they are suffering from, it makes one aware what kind of a problem they have.’ (IDI 21, interview 21, 45 years, single, 2 children)

One participant was satisfied with the information she was getting and hoped it would continue as it was motivating her to screen. She further suggested that free treatment for cancer would motivate more women to undergo screening:

‘I wish that the way we are being given information would continue and not stop, and also if one is found to be having the disease one could have access to treatment, just as it is being done for HIV [*human immunodeficiency virus*], that one is treated for free. What would make it worse is when one has cancer of the mouth of the womb and they have no money to buy the prescribed medication.’ (IDI 6, interview 6, 40 years, married, 2 children)

## Methods of information dissemination

Participants pointed out various methods of disseminating information. They suggested that health workers should go out into the community and educate women on cervical cancer, strategies such as door to door campaigns, pitching up tents and screening women and also going into churches and teaching people about the disease:

‘I think if there is a door-to-door campaign so that people have information, if you educate a woman she will have knowledge that there is such a thing rather than her going to the clinic. Sometimes at that clinic they won’t be having those facilities so they don’t talk about it.’ (FGD1, P1, 10 January 2018)

‘I think if they go to various places and pitch up tents and test women, I think it will help because women are afraid. I think also when women go to deliver they should be tested for cervical cancer before delivery.’ (IDI 20, interview 20, 35 years, separated, 2 children)

‘I think by going where many people gather like churches and be taught on the signs and symptoms. If people become aware of the symptoms and once they see these they will then rush to go to the hospital.’ (IDI 13, interview 13, 54 years, single, 1 child)

## More screening centres and combining screening with HIV care

Integrating services of HIV and cervical cancer screening was suggested a solution to increasing cervical cancer screening numbers. Women suggested that it would be convenient to access all services under one roof. Other participants pointed out that there was need to avail more centres for screening as the ones currently available were far and few:

‘I think that if there could be a cervical cancer screening clinic here it would make it easier for women to undergo screening, so that when one comes for review they can screen for cervical cancer. There is need for the whole body to be assessed, so that one becomes aware of their health status. I may be moving around and yet I could be rotten.’ (FGD4, P1, 21 February 2018)

‘If it could be made more accessible by screening women at the local clinics. Because going into town to book or other far off area, I may not have the money for transport, but if they say on such and such a day women should go to the clinic for screening. If I feel like screening I will then go.’ (IDI 2, interview 2, 47 years, single, no child)

‘I think there should be more centres for screening so that women are not put off by the long queues, one can be put off by the thought of spending the whole day in the queue. There should be more centres such that when one feels like screening they just walk into a centre be screened and walk out.’ (FGD2, P1, 21 February 2018)

## Free treatment for cervical cancer

Generally, participants pointed out that treatment for cervical cancer was too expensive, by making treatment free it was suggested this would encourage more women to undergo screening. Participants opined that because HIV care was free, treatment for cervical cancer should be free of charge as well. Removing this barrier would motivate more women to be screened:

‘I would just like to appeal to the ministry of health to make treatment for cervical cancer to be free, once they do that more women will be forthcoming and they would save a lot of lives. A lot of women are suffering from cervical cancer and are at home, because when they think of treatment and the charges they give up as it is expensive.’ (FGD3, P3, 6 February 2018)

‘I wish that people would have access to treatment as it is being done for HIV [*human immunodeficiency virus*], that one is treated for free. What would make it worse is when one has cancer of the mouth of the womb and they have no money to buy the prescribed medication.’ (IDI 4, interview 4, 45 years, married, 3 children)

‘If one has no money for treatment they should not be screened for cervical cancer, it is best to let it be.’ (FGD4, P4, 21 February 2018)

It was also highlighted that a lot of women will die because of failure to access cancer treatment because of the high cost. Some of the participants highlighted that they are willing to screen but the high cost of treatment is an obstacle:

‘Truly speaking, if they make cancer treatment free a lot of people will go for screening.’ (FGD4, P3, 21 February 2018)

## Discussion

The study revealed that facilitators to cervical cancer screening can be viewed as cues to action in the health belief model. Cues to action can be events, people or things that spur people to change behaviour (Orji, Vassileva & Mandryk 2012:6). In this study some women felt that being HIV-positive made them more vulnerable to cervical cancer as such this facilitated the screening behaviour. Bukirwa et al. (2015:9) also found that the motivation to screen by women in a study in Uganda was the perceived risk because of HIV status and being sexually active.

The study showed that having signs and symptoms motivated women to screen for cervical cancer, the results are similar to a study by Bateman et al. (2018:3), which found that women were motivated to screen by experiencing symptoms and being told by a counsellor to seek screening. However, the participants in this study were aware that early screening and detection were important for a better outcome. Similarly, the main reason for not screening for cervical cancer in a study carried out by Bayu et al. (2016:9) was the absence of symptoms, moreover this perception is concerning because by the time the symptoms appear the benefits of screening have likely been missed. Such perceptions bring forth the need to raise awareness about cervical cancer screening amongst women.

For some women importance of screening for cervical cancer in terms of saving one’s life overrides concerns about discomfort of the procedure. Some women indicated that they had young children and hence needed to know that they were safe. O’Connor et al. (2014:478), found that women felt personally responsible for their health and some felt having smears provided them with reassurance.

Receiving information from counsellors’ motivated women to screen in this study. It has been shown that attitude of health workers with respect to screening and women’s health issues has a major influence on women’s attendance for screening. Fletcher et al. (2014:5), opined that participation in cervical cancer screening would improve if women had strong relationships with healthcare providers.

Some of the participants suggested that women should be forced to go and screen, and that it should not be left up to them to decide to; it should be mandatory. While information has been found to be the gateway to initial participation in screening for cervical cancer regular reminders are required for continued participation. Women also suggested that they needed reminders to screen and information about cervical cancer: women in a study by Addawe, Mburu and Madar (2018:3) suggested use of invitation letters to all women so that they could attend for screening.

Participants highlighted that the information received was insufficient and they had only been informed to go for screening without much detail about the disease. In a study by Ports, Reddy and Rameshbabu (2015:100), women expressed that health information facilitated their behaviours, they thought that more information on cervical cancer would encourage screening. Binka, Doku and Awusabo-Asare (2017:12) suggested that thorough mass media and community health education can be employed to create awareness about cervical cancer.

Participants suggested that health workers should go out into the community and educate women on cervical cancer, embark on door-to-door campaigns, go to churches and pitching up tents to teach people about cervical cancer. In Malawi many women wanted to be community advocates who wanted to participate in and be responsible for educating others in the community, other women indicated that they would urge others to go to hospital and get information from nurses and doctors about cervical cancer (Ports et al. 2015:101). They pointed out that nurses can play a vital role in persuading women to undergo screening. Kenya et al. (2015:264) also found that women preferred one on one conversations in clinic waiting rooms.

Participants suggested that television and radio were valuable media, which can be utilised to increase participation in cervical cancer screening services. Ebu et al. (2015:36) suggested that education on cervical cancer through mass media is important in informing women about cervical cancer and the facilities available for them.

Peer health education has been found to be an effective strategy for increasing women’s perception of benefits of early detection of cervical cancer through screening and also increasing their practice of screening for cervical cancer. Participants highlighted that they benefitted from discussions with their peers. Kiminski (2011:4) stated that ‘the concept of peer education draws on the diffusion of innovation behavioural theory where innovators and early adopters serve as opinion leaders’, these individuals act as agents of change by disseminating information and influencing their peers in their community.

Some of the women suggested that it would be more convenient for them to access all services under one roof. They were of the opinion that integrating HIV and cervical cancer screening services would be ideal. The lack of access to healthcare as a result of unavailability of screening sites and long distance to the facilities negatively impacted the screening behaviour of women (Ebu et al. 2015:37). It has been suggested that it is feasible to integrate cervical cancer screening with HIV care (Bekolo et al. 2016:8). Regular attendance of HIV treatment centres impacts the awareness of the risk of cervical cancer but it still has no effect on screening behaviour.

One of the reasons put forward was that ‘physicians responsible for monitoring and treatment of HIV patients do not have sufficient training to counsel patients on the prevention of cervical cancer’ (Sichanh et al. 2014:8).

Participants in the study pointed out that treatment for cervical cancer was too expensive, even though screening for cervical cancer is free they stated that cost of treatment is a barrier to screening. They suggested that cervical cancer treatment should be free just as HIV treatment. Andreassen et al. (2017:52) found that almost everyone in their study stated that free screening would be a very good thing and if people knew about it more women would participate. In an effort to motivate women to take up screening and treatment, these services should be made affordable if not completely free.

## Limitations

In-depth interviews and focus group discussions yielded rich qualitative data but it cannot be generalised as data were collected from only one health facility, this could have limited different views from other women in other health facilities. To closely examine the differences between adopters and non-adopters of cervical cancer screening required use of both quantitative and qualitative methods to form a larger more representative sample. The study did not investigate perspectives from healthcare providers and policymakers, which would have provided important information regarding the logistical barriers and facilitators of cervical cancer screening programmes.

## Recommendations

The study recommends development of clear and simple educational messages about cervical cancer and screening that can easily be understood by women and these messages should be communicated appropriately. Increase in information outlets in the communities to raise awareness and improve knowledge, such as in churches, beerhalls and shopping malls. Developing programmes such as peer education, which can increase awareness about cervical cancer in HIV-positive women.

## Conclusion

The study revealed that there should be more screening centres for easier access, integration of cervical cancer screening into HIV-care and more facility-based health education on cervical cancer and screening. There is need to improve information dissemination by healthcare providers through door-to-door campaigns and going into churches as well. Provision of free treatment could be a solution to improve screening rates. Furthermore, to facilitate screening there is need to publicise cervical cancer screening services through use of mass media such as radio and television. Provision of affordable and quality care may also go a long way in improving uptake of screening.
